# Textile-Based Electronic Components for Energy Applications: Principles, Problems, and Perspective

**DOI:** 10.3390/nano5031493

**Published:** 2015-09-07

**Authors:** Vishakha Kaushik, Jaehong Lee, Juree Hong, Seulah Lee, Sanggeun Lee, Jungmok Seo, Chandreswar Mahata, Taeyoon Lee

**Affiliations:** Nanobio Device Laboratory, School of Electrical and Electronic Engineering, Yonsei University, 50 Yonsei-ro, Seodaemun-Gu, Seoul 120-749, Korea; E-Mails: vishakhakaushik@gmail.com (V.K.); sotage2@gmail.com (J.L.); juree@yonsei.ac.kr (J.H.); esa0605@yonsei.ac.kr (S.L.); sang0927@yonsei.ac.kr (S.L.); jungmok.seo@gmail.com (J.S.); chandreswar@gmail.com (C.M.)

**Keywords:** wearable electronics, textile electronics, conductive fabric, textile sensors, energy harvesting and storage

## Abstract

Textile-based electronic components have gained interest in the fields of science and technology. Recent developments in nanotechnology have enabled the integration of electronic components into textiles while retaining desirable characteristics such as flexibility, strength, and conductivity. Various materials were investigated in detail to obtain current conductive textile technology, and the integration of electronic components into these textiles shows great promise for common everyday applications. The harvest and storage of energy in textile electronics is a challenge that requires further attention in order to enable complete adoption of this technology in practical implementations. This review focuses on the various conductive textiles, their methods of preparation, and textile-based electronic components. We also focus on fabrication and the function of textile-based energy harvesting and storage devices, discuss their fundamental limitations, and suggest new areas of study.

## 1. Introduction

**Figure 1:**
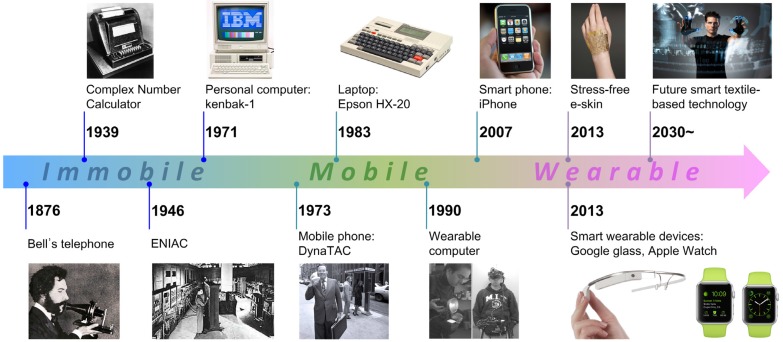
A chronological illustration presenting the journey of electronic components from immobile to mobile, and finally to wearable textile-based electronics.

Electronic devices have the ability to address basic needs [1,2,3,4,5,6]. When electronic devices were first being developed, scientists imagined great leaps forward such as the development of artificial intelligence, and realized the innovation of the Bell telephone in 1876 [7]. A complex number calculator was later developed by the Bell laboratory in 1939 [8]. Seven years after this valuable development, the first fully-functional digital computer, known as ENIAC, came into existence [9]. Although ENIAC was the fastest machine at that time, it was difficult to physically transport due to size (2.4 × 0.9 × 30 m^3^) and power requirements (150 kW). Thus, the primary goals of researchers at this time were to decrease the size and power consumption of electronic devices, which they did with the miniaturization of electronic devices and developed the first personal computer, Kenbak-1, and the first laptop computer, Epson HX-20 [10,11,12]. The increasing pace of technological developments since the late 20th century has resulted in exploration of mobile and wearable devices [13,14]. Early stage wearable computers were composed of backpack systems with uncomfortable wiring networks. Currently, researchers aim to develop flexible wearable devices that integrate seamlessly into regular clothing such as Google glass, the Apple watch, and electronic skins [15,16,17,18]. An illustration of the history of these electronic components from Bell’s telephone to smart wearable devices is presented in Figure 1 [7,8,9,10,11,12,13,14,15,16,17,18,19]. Recent market demand has focused on small electronic components for smart wearable device applications, leading to research and products featuring technology that can directly integrate electronic components into textiles or fabrics for a variety of applications including sensors, medical devices, safety instruments, and planar waveguides [4,20,21,22,23,24,25,26,27,28,29,30,31].

Textiles have been used by mankind since prehistoric times, and they are essential to daily life. Fashion dictates that the textiles must look attractive and fit comfortably. Traditional textiles are composed of natural or synthetic fibers and filaments. Recent advances in electronics have led to the development of a new generation known as electronic textiles (E-textiles) or smart textiles. E-textiles consist of fabric that provides advanced functions in the form of electronic features or interconnection [32,33]. E-textiles can be categorized into two types: classical, where the electronic components are embedded into garments and integrated, where the electronic components are directly integrated into textile substrates. E-textiles have attracted attention in technical and socioeconomic fields because of their ability to sense and respond to environmental stimuli [33,34]. Due to this ability, they are ideal components for smart wearable electronics, and represent a new branch of flexible multifunctional fabric electronics. Many research groups have explored the integration of flexible devices such as solar cells, field-effect transistors (FETs), light emitting diodes (OLEDs), and photovoltaic devices into textiles; however, these components cannot operate without sufficient electrical energy, making the development of energy harvesting and storage devices essential [35,36,37,38,39,40,41,42,43,44,45,46].

This paper reviews the development of various key components that are required for E-textiles. The most fundamental element is the conductive textile, which is required for interconnection to transfer power and data. Energy harvesting and storing applications are necessary for powering the electrical components such as transistors, LEDs, and sensors for their utilization in various functional E-textiles. A systematic view is suggested and elucidated with examples.

## 2. Conductive Textile: Materials and Fabrication

It is ideal for electronic interconnects to be realized on fibers or fabrics for the fabrication of textile-based wearable devices. The development of highly-conductive textiles for interconnection is crucial to minimize energy loss. Three types of materials have been explored for the fabrication of high-conductivity textiles: polymer-based, carbon-based, and metallic materials. In this section, the features of conductive textiles consisting of organic and inorganic materials will be discussed.

### 2.1. Polymer-Based Conductive Textiles

Conductive polymers have attracted significant scientific interest for their interesting and unique electrical, optical, and mechanical properties [47,48,49]. They have been explored in a number of applications such as chemical and biological sensors, drug delivery, corrosion protection, nanoelectronic devices, biomedicine, microwave absorption, and electrocatalysis [50,51,52,53,54,55,56,57,58,59]. Shirakawa *et al.* were the first to demonstrate conductivity in polymers via doping, showing that exposure of polyacetylene (PA) polymer to iodine vapor increases conductivity by up to seven orders of magnitude [60]. The enhanced conductivity of PA is attributed to redox reactions (charge transfer complexes) between the PA and iodine vapor. The search for highly conductive polymers has resulted in the expansion of this area, and a number of conductive polymers have been used in the last 35 years. Various applications such as protective clothing, sportswear, health monitoring, actuators, and sensors for wearable E-textiles have now been explored via these conductive polymer fabrics [61,62,63,64,65].

**Figure 2:**
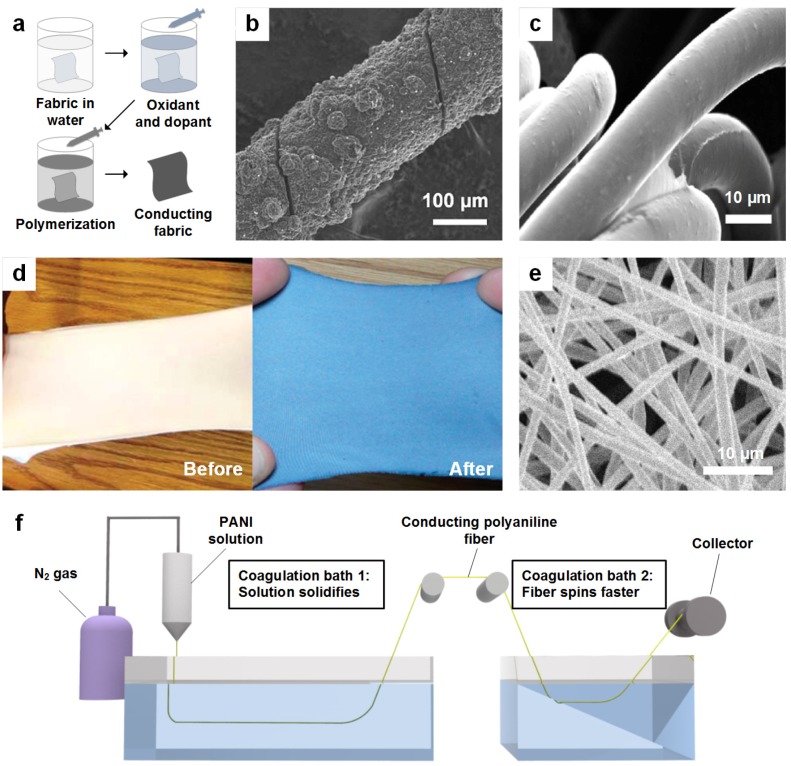
(**a**) Schematic illustration of the *in situ* chemical polymerization for conducting polymer-coated fabric; (**b**) SEM image of a conductive fiber composed of carbon nanotube bundles coated with polypyrrole; (**c**) SEM micrograph of stretchable spandex fabric after a dip coating process with polystyrene sulfonate-doped poly-(3,4-ethylenedioxythiophene) (PEDOT:PSS); (**d**) Optical image of stretchable fabric before and after dip coating with PEDOT:PSS; (**e**) Surface morphology of polyaniline (PANI): polyethylene oxide (PEO) electrospun fiber; (**f**) Wet fiber spinning technique process. Reproduced from [68,73,74] with the permission by Royal Society of Chemistry, Copyright 2015, and by ACS Publications, Copyright 2010, 2012.

Conjugated polymer polypyrrole (PPy) has received much attention due to its high conductivity paired with chemical and environmental stability [66,67]. PPy also has the advantage of being easy to synthesize at large areas with different porosities at room temperature. Conductive polymer textiles can be fabricated via polymerization, wet spinning, or dip coating processes [68,69,70]. *In situ* polymerization or chemical oxidation polymerization are commonly used [71,72]. The experimental steps used to fabricate conductive PPy via in-situ chemical polymerization are illustrated in Figure 2a. Highly-conductive PPy nanofibers with diameters between 60 and 100 nm that are synthesized in p-hydroxyazobenzene sulfonic acid act as a functional dopant. PPy is deposited when the fabric and polymer solutions come into contact due to the liquid-solid interface interaction. This is considered a physical adsorption followed by a polymerization process, and results in a layer of conducting polymer with a smooth surface over the fibers. PPy can also be deposited onto other complex structures. Figure 2b shows the scanning electron microscope (SEM) image of a conductive fiber composed of carbon nanotube bundles coated with PPy [73]; however, brittleness and rigidity are two potential drawbacks to using PPy.

Another interesting conductive polymer is a polythiophene derivative, poly-(3,4-ethylenedioxythiophene) (PEDOT), which shows high electrochemical stability in oxidized form due to its planar structure and delocalized π electrons [75]. Its unique structure contains dioxyalkylene bridging groups at positions 3 and 4 of its heterocycle ring, making it the most valuable among all conducting polymers. Thus, it shows high conductivity and good electrical, thermal, and chemical stability when compared to PPy. Although PEDOT itself is a conductive polymer, it has low solubility, which limits usage. Generally, polystyrene sulfonate (PSS)-doped PEDOT (PEDOT:PSS) is used to obtain a stable dispersion in a water solvent. PEDOT:PSS has been used extensively in the last few years as a conducting polymer [76]. The simplest technique for coating is dip coating, where the fabric is simply dipped into the conductive solution, resulting in a conductive polymer fabric. Ding *et al.* have reported the preparation of PEDOT:PSS conductive fabrics using the dip coating process [68]. Figure 2c shows the surface morphology of a spandex fabric after soaking in a commercially-available PEDOT:PSS dispersion. It is clear from the Figure 2c that the surface of the fabric is smooth and uniform after conductive coating. The conductivity of the single soaked fabric was reported to be 0.1 S/cm, reaching up to 2 S/cm after multiple soaking steps. The optical image of the PEDOT:PSS-soaked textile is shown in Figure 2d. Thus, conductive fabrics can be produced in a simple manner.

Polyaniline (PANI), known as aniline black, has also been studied as a conductive polymer. PANI revolutionized polymer chemistry due to its many advantages including stability, cost effectiveness, and switching characteristics between conductive and resistive states. Its electrical conductivity is due to the partial oxidation or reduction process and can be tuned to achieve the required conductivity for a given application. It is fabricated via a chemical oxidative polymerization process of aniline, which is a multistep and slightly complex process. Figure 2e shows the surface morphology of electrospun PANI fibers that exhibit high electrical conductivity between 70 and 150 S/cm [74]. However, a more simplified, single-step fabrication method is reported by Pomfret *et al.* where polyaniline fibers are fabricated using a wet spinning process [77]. The resulting fibers exhibited high conductivity (~1000 S/cm for bulk material) and good mechanical strength (young modulus ~2 GPa). Figure 2f shows the schematic design of the wet spinning process utilized for the production of conductive polyaniline polymer. A mixture of organic solvents and the emeraldine base form of polyaniline is used to make a spinning solution, which then enters the first coagulation bath where it solidifies and forms fibers. In the second coagulation bath, the fibers spin rapidly, resulting in stretching. The stretched fibers are then doped with an acid suitable for the preparation of conducting fibers, as shown in Figure 2f. As discussed earlier, conductive polymers are considered useful in wearable textiles; however, the conductivity values should be modified for their optimization in the production of smart wearable electronics. A comparison of the properties of these conductive fibers is given in Table 1.

**Table 1:** Summary of materials and their electric properties.

Structure of conductive textile	Material use to prepare or to coat on fibers	Merits of Conductive textiles	Technique used to fabricate conductive textile	Advantages of growth technique	Disadvantages of growth technique	Electrical property	Ref.
**Polymer-based**	Polypyrrole	Chemical and environment stable	Functional dopant induced process	Novel & Easy process, High Yield easily deposit on fabric surface	Need to optimize dopant concentration for better conductivity results	120–130 S/cm	[78]
	Polyaniline	Cost effective, stable	Wet Spinning	Produced fibers show high electronic and mechanical strength, fabricated thick fibers	Need to combine Individual fibers	140-750 S/cm	[79,80,81]
	PEDOT:PSS	Highly conductive good thermal and chemical stability	Dip Coating	Simple process	Coating is slow, tide lines can forms	0.4–2.0 S/cm	[68,82]
**Carbon-based**	Graphene	Highly conductive & stable, high strength	Electrostatic self-assembly with BSA followed by chemical reduction	Easy to attach graphene oxide films to textiles	Multistep process, wrinkles observed on surface of samples	10–20 S/cm	[83,84]
	Carbon nanotubes	Conductive, high mechanical strength	Wet spinning	Continuous long length (cm) CNT yarns	Slow processing	125–3000 S/cm	[85,86,87]
**Metal-based**	Al-coated conductive paper	Stable, conductive, stretchable	Chemical solution process	Low cost, Vacuum not required, Direct deposition of Al on fibers	Need a suitable catalyst for decomposition process	19 mΩ/□	[88]
	AgNP/Ag nanowires	Conductive	Wet spinning	Simple, highly conductive material	Need optimization of suitable solvents and coagulation liquids	2200–5400 S/cm	[89,90]

### 2.2. Carbon-Based Conductive Textile

**Figure 3:**
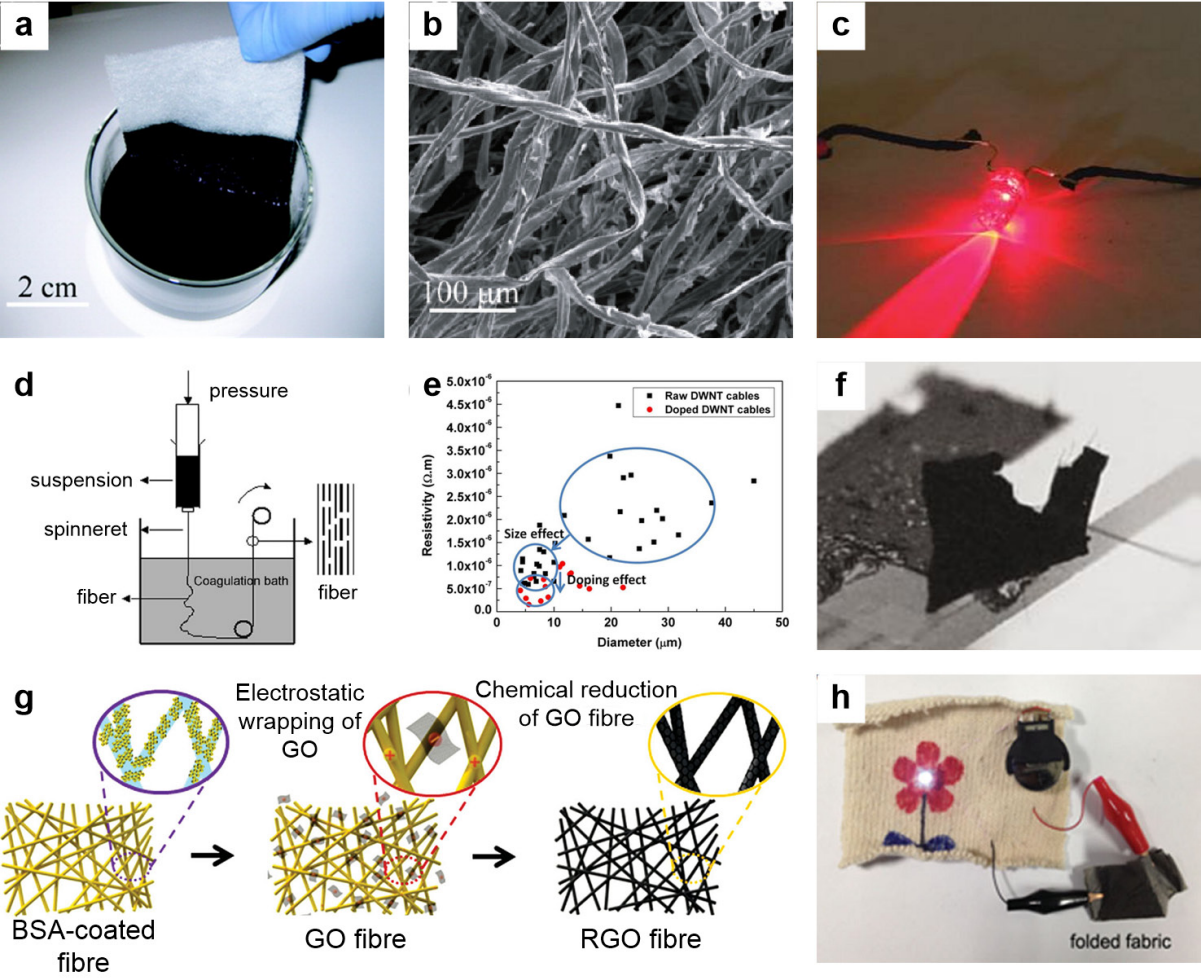
(**a**) Optical image shows the dip-coating process of fabric into single-walled carbon nanotube (SWCNT)s ink for SWCNT-based conductive fabric; (**b**) SEM image of a cotton sheet coated with SWNTs on the fibrous surface; (**c**) Demonstration of LED emission with CNT-based conductive fibers; (**d**) Schematic of the experimental setup of the wet spinning method for CNT-based conductive fibers; (**e**) Resistivity *versus* diameter plot for raw DWNT (double walled CNT) and iodine doped DWNT cables; (**f**) Image shows a free standing CNT array through which a yarn is pulling out; (**g**) Schematic illustration of the fabrication process of graphene oxide-based conductive fiber using electrostatic force; (**h**) Photograph of a LED light using a folded, reduced graphene-oxide-based conductive fabric. Reprinted from [83,87,102,103,104,105] with the permission by ACS Publications, Copyright 2010, 2008 and by John Wiley and Sons, Copyright 2008, 2013 and by Nature Publishing Group, Copyright 2002, 2011.

Carbon-based materials including carbon nanotubes (CNTs), carbon fibers, carbon nanoparticles, and graphene present extraordinary properties such as high mechanical strength, light weight, environmental stability, and superior thermal and electrical conductivity [91,92,93,94,95,96,97,98]. These intriguing properties make carbon-based materials important candidates in wearable electronic textiles. Among all carbon allotropes, the most widely explored are CNTs and graphene. While carbon fibers have been explored in the textile industry for a long time, CNTs are increasingly chosen over commercial carbon fibers because of their lower density, and higher tensile and compressive strength [99,100,101]. CNT can be coated on various fibers such as cellulose or polyester yarns via a simple and cost effective dipping and drying method, as shown in Figure 3a [87]. Here, cotton fibers are simply dipped into a single-walled carbon nanotube (SWCNT) solution, resulting in a black conductive fabric. Figure 3b shows the surface morphology of the SWCNT-coated cotton fibers. The as-prepared CNT-cotton yarns show high electrical conductivity (125 S/cm) with outstanding stretchability and flexibility. The coating of CNTs results in conductivity of fibers and a CNT-cotton thread can emit the light of an LED, as shown in Figure 3c [102].

CNT fibers without any supportive fiber can be made using wet or dry spinning methods. Zhang *et al.* reported a wet spinning method for the production of long CNTs; a schematic of their wet spinning process is shown in Figure 3d [103]. The electrical conductivity of CNT fibers was enhanced up to ~6.7 × 10^4^ S/cm by iodine-doping [104]. The enhancement in the conductivity of CNT fibers via doping is shown in Figure 3e [104]. Jiang *et al.* have fabricated CNT yarns using a super-aligned array of CNTs [105]. The way to produce continuous CNT yarns 30 cm in length from a CNT array is similar to drawing a thread from a silkworm cocoon. Figure 3f shows an optical image of the yarn pulled from a CNT array [105]. The authors have constructed a light bulb filament using these CNT yarns and observed that their strength and conductivity is enhanced by heating at high temperatures. The CNT-yarn-based filament emits incandescent light for 3 h, and after this time span the conductivity of the filament increases by up to 13%, while the tensile strength of the CNT yarn increased from 1 mN to 6.4 mN. Thus, these CNT yarns can be woven into fabrics [105]. However, this process has some disadvantages in terms of slow processing and limited to scaling for thick or multistrand forms. Other issues include high junction resistance.

Graphene oxide (GO) has also been widely studied for conductive fibers. Yun *et al.* presented an electrostatic method for fabricating textiles that are wrapped with reduced graphene oxide (RGO), which makes them conductive and stable (Figure 3g) [83]. Nylon-6 yarns were functionalized with bovine serum albumin molecules to obtain positively-charged surfaces. GO sheets were then electrostatically assembled over the fibers via a simple immersing and shaking process in a GO solution [83]. Finally, the GO are chemically reduced to RGO. They have also demonstrated the lightning of an LED using the synthesized conductive fibers, a process that works well even after folding, as shown in Figure 3h [83]. Freestanding RGO fibers can also be synthesized using wet or dry spinning methods. Xu *et al.* have reported porous graphene oxide with good electrical conductivity (~4.9 × 10^3^ S/m) and high specific surface area (884 m^2^·g^−1^) [106]. This graphene-based hybrid structure presents great potential in fiber-based electrochemical super-capacitors [84]. The above described properties of carbon-based materials open a new path in electronic textiles for wearable electronics. However, further investigation is needed to look into the possible side effects of wearable electronics, such as toxicity with skin contact and stability in air.

### 2.3. Metal-Based Conductive Textiles

Metallic materials are considered a better choice for wearable electronics because of their high conductivity [89,90]. Vacuum deposition is one of the most widely used techniques for metal deposition and is also frequently used for microfabrication processes. Sputtering or evaporation methods can be used to directly form a thin metal layer on the exposed surface of the fibers [107,108]; however, these techniques have huge drawbacks such as expensive instruments, limited sample size, and non-compatibility for batch process. Also, these methods are not suitable for depositing metal on complex structures such as porous fibers, and the deposited films are weak against repeated deformations like folding or bending. In order to avoid these issues, alternative methods must be used to synthesize metal-based conductive textiles.

**Figure 4:**
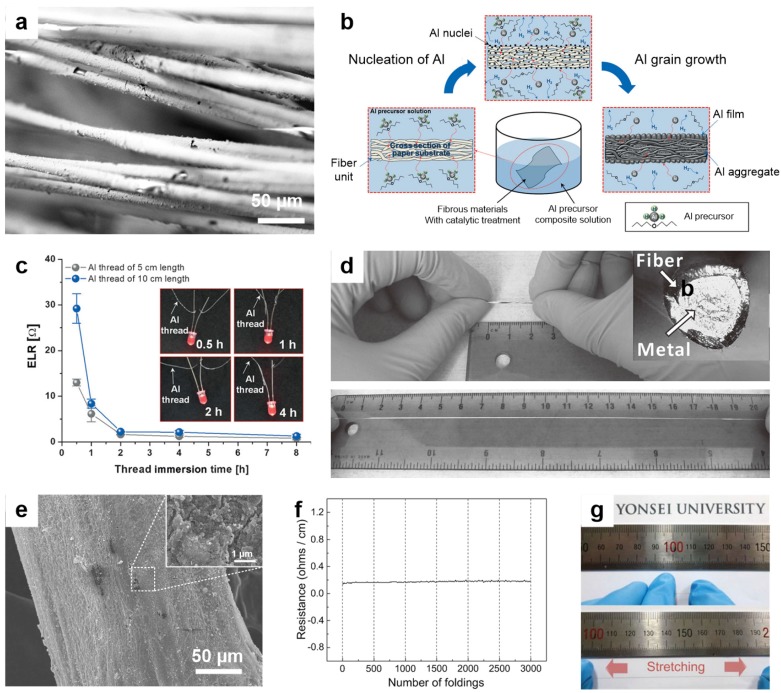
(**a**) SEM image of conductive Kevlar fibers coated with Ni/Au; (**b**) Schematic representation of the chemical solution process for the fabrication of conductive Al textiles; (**c**) Plot of the electrical linear resistance of Al-based conductive fiber synthesized by the chemical solution process and inset shows the functioning LEDs; (**d**) Optical image of unstretched and stretched (up to 20 cm) conductive fiber; (**e**) SEM image showing the surface of the Ag nanoparticles and elastomer composite-based conductive fiber; (**f**) Electrical resistance change in the Ag-based conductive fiber during the 3000 cyclic folding test; (**g**) Optical image of pre- and 900%-strained Ag nanoparticles and elastomer composite fibers. Reproduced from [20,88,90,109,112] with the permission by ACS Publications, Copyright 2011 and by John Wiley and Sons, Copyright 2013, 2013, 2015, 2015.

Electroplated or electroless plated metals, liquid metals, and metallic nanomaterials such as nanoparticles or nanowires are considered ideal candidates in the growing field of wearable electronics [109,110]. Metal plating methods have been widely used to deposit metal for printed circuit boards, metallization processes in microfabrication, and corrosion protection. Figure 4a shows an SEM image of a gold-plated conductive fiber, in which Kevlar fibers were first treated with Sn^2+^ and Pd^2+^ ions followed by electroless Ni deposition for conductivity [109]. Au was then electroplated onto the surface to achieve a conductive fiber with a conductivity of ~6 S/cm and power output of 1 W. However, the metal films are weakly attached to the surface and may separate from the fiber during further processing. To overcome these obstacles, a recent study presents a new, simple, and inexpensive chemical solution method, as shown in Figure 4b [88]. In this process, an Al precursor was used to deposit Al on the surface of fibrous materials. Figure 4c shows the electrical resistances verses immersion time in the Al precursor solution, and the inset shows an LED connected to these fibers [88].

Another method of synthesizing metal-based conductive textiles is the use of liquid metals [111]. Eutectic gallium indium (EGaIn) is a liquid metal with high conductivity (σ = 3.4 × 10^4^ S/cm) and a low melting point (*T_m_* = 15.7 °C) that spontaneously forms a thin oxide layer that can be used to mold the metal into complex shapes [111]. When sufficient force is applied, the oxide layer can be damaged and the shape of the EGaIn can be changed. Zhu *et al.* has filled a hollow elastomer fiber with EGaIn to obtain stretchable conductors, as shown in Figure 4d [112]. The authors observed that the metallic continuity is maintained even after the applying a 700% strain.

Various metallic nanomaterials such as silver, copper, aluminum, gold, and tin are used as conductive materials for textiles [113]. Atwa *et al.* demonstrated nylon, polyester, and cotton threads dip-coated with Ag nanowires (AgNWs), which exhibit high mechanical flexibility and a small increase in resistance after 200 bending cycles as compared to commercial conductive fibers [113]; however, metallic nanomaterials mixed with polymers can lead to better stability. Recently, Lee *et al.* reported the fabrication of silver nanoparticle (AgNP)/polymer-coated Kevlar fiber with a silver content of more than 80 wt % [20]. Figure 4e shows the surface morphology of the as-prepared fibers along with an inset showing the AgNPs on the surface [20]. In this work, the AgCF_3_COO solution was absorbed by a poly (styrene-block-butadienstyrene) (SBS) polymer and then reduced to AgNPs using hydrazine hydrate. Figure 4f shows the small change in resistance during 3000 folding tests [20]. In another study, Lee *et al.* inserted AgNWs into a stretchable AgNP/SBS structure to obtain increased conductance under strain [90]. The fiber shows high electrical conductivity, 2450 S/cm, and can stretch up to 900% strain as shown in Figure 4g [90]. Table 1 presents a comparison of the properties of these metallic conductive fibers.

## 3. Textile Electronic Components

Textile-based electronic devices require electronic functionalities on fiber/textile materials with robust mechanical properties. In this section, we will discuss electronic devices based on fibers and their use in textile electronics. The operation of electronic devices and their stability in a textile system will be explored in detail. Specifically, we will demonstrate recent developments in textile-based electronic devices such as transistors, light emitting diodes (LEDs), and sensors.

### 3.1. Transistor

The integration of electronic devices into textiles at the fiber level is vital to the future of wearable electronics. There are various aspects to be considered before employing electronic devices into a textile-based system such as materials, structures, and the compatibility of electronic devices with textile fabrics. As a fundamental building block of electronic devices, transistors must be integrated into fabric or textiles. In this section, we briefly introduce fiber or textile-based transistors and their operation. In general, an electronic device that can amplify and convert electronic signal to electrical power is known as transistor. Since transistors are basic components in electronic devices, the integration of transistors into fabric or textile systems is highly important. However, conventional inorganic FETs cannot be applied to fabric or textile-based electronics due to flexibility issues and limitations in the fabrication process. Given the technological limitations of inorganic materials, organic thin film transistors (OTFTs) have received significant attention owing to their flexibility, compatibility with flexible electronics, light weight, biocompatibility, low cost, and low-temperature process capability [114,115,116,117].

**Figure 5:**
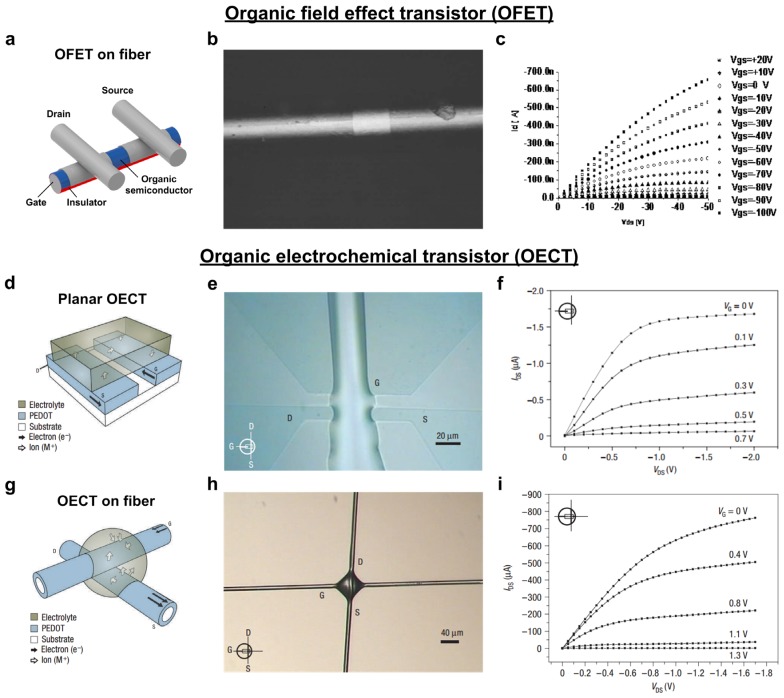
(**a**) Schematic picture of the organic field effect transistor (O-FET); (**b**) Optical image showing channel area; (**c**) The output characteristic of O-FET; (**d**) Schematic picture of a planar OECT; (**e**) Optical micrograph of a planar organic electrochemical transistor (OECT); (**f**) Output characteristic of a planar OECT; (**g**) Schematic picture of a fiber OECT; (**h**) Optical micrograph of a fiber OECT; (**i**) Output characteristic of a fiber OECT. Reprinted from [122,123] with permissions by AIP Publishing LLC, Copyright 2006 and by Nature Publishing Group, Copyright 2007.

Organic-based transistors due to their flexibility, easy fabrication process and comparable electrical performance (as compared to inorganic TFTs) are desirable in textile industry [118,119,120]. Two types of fiber-based OTFTs have been reported; fiber organic field effect transistors (F-OFETs) and organic electrochemical transistors (OECTs) [121]. The working principle of F-OFETs is similar to that of commercial FETs. In O-FETs, organic thin-film semiconductors are used as an active layer instead of inorganic materials. Figure 5a shows a schematic diagram of F-OFETs. Here, a commercially available cylindrical metal fiber (Al & stainless steel) is insulated using a uniform layer of polyimide, followed by the evaporation of pentacene as an active material PEDOT:PSS is then deposited on the pentacene surface using a soft lithographic process to form source and drain electrodes. Figure 5b,c shows an optical microscope image of the channel length (~45 µm) and corresponding *I*_D_-*V*_D_ curve of an F-OFET [122]. In spite of the low value of the width/length ratio of the F-OFET, the on current and on-off ratio are reasonably high (~10^4^), which is fully comparable with those of planar devices. Although F-OFETs exhibit decent electrical properties, they suffer from significant disadvantages such as high operating voltages, high equipment cost, and a complicated fabrication process. OECTs have been considered as an alternative to F-OFETs, since they can be fabricated on the basis of fiber structure. The working principle of OECTs is based on the electro-chemical process, where a reversible process of doping and de-doping of electronic polymers allows the transistor to operate. Figure 5d shows a schematic picture of OECTs on a planar substrate [123]. Here, source (S), drain (D), and gate (G) electrodes are demonstrated on flat substrates such as glass, plastic, and paper. The conducting polymer PEDOT/PSS films are first micro-patterned on a flat substrate, and are followed by the pattering of an electrolyte (micro-channel of ~3 µm height and ~15 µm width) over the patterned PEDOT/PSS film.

An optical micrograph of this configuration is shown in Figure 5e, and the corresponding output characteristic of a planar ECT is shown in Figure 5f [123]. The output characteristics clearly indicate that the current is saturated with increasing drain voltage, and that the gate voltage is low compared to that of F-OFETs. When the gate voltage is zero, the transistor is in the ON state, while the gate voltage transistor moves to the OFF state when the gate voltage is further increased, as shown in Figure 5f. In order to fabricate OECTs on a fiber structure, a simple new method was performed by Hamedi *et al.* [123]. Figure 5g shows a schematic picture of a fiber OECT constructed using PEDOT-coated polyamide fibers (~10 µm diameter). Two PEDOT-coated fibers are kept in a cross geometry and an electrolyte is dropped at the fiber junction, as shown in the optical image in Figure 5h [123]. In this cross geometry, one PEDOT-coated fiber acts as source and drain and the other as gate electrode. An active material (electrolyte with 33 wt % PSS, 12 wt % glycol, 8 wt % sorbitol, water and 0.1 M NaClO_4_) acts as a conducting channel, which is controlled by the gate electrode. In this way, micrometer-sized fiber OECTs can be easily integrated into textiles. The output characteristics of fiber OECTs are similar to planer OECTs, as shown in Figure 5i [123]. The similar electrical characteristics between fiber OECTs and planer OECTs are attributed to the fact that the operation of electrochemical transistor is dominated by the interface effect between the conducting polymer and electrolyte. Therefore, fiber OECTs can be used as effective transistors in spite of completely different geometry when compared to planar OECTs. Theoretically, the fiber OECTs could be used for large scale production: ~100,000 transistors/cm^2^ can be constructed using 10 µm fibers. Also, the authors have fabricated a binary tree multiplexer structure by using these fiber OECTs. This multiplexer is able to encode the information from a large number of data sources into a single channel. Hamedi *et al.* fabricated a fiber-based OECT using poly 3-hexylthiophene (P3HT) with 100 µm channel length and showed that the fiber can be operated below ~1 V [124].

Since fiber OECTs have many advantages such as low operating voltages, large amounts of current densities, and low cost production, they can easily replace OFETs and can be integrated to wearable electronics by directly weaving them into fabrics. Bonderover *et al.* have demonstrated textile inverters made by weaving fiber-based transistors [125]. They have shown that E-textiles with electronic components can be designed by simply woven fibers. Thus, in the future it is possible to fabricate such E-textiles. However, their long response time and low switching frequency remain significant hurdles. In addition, the exact charge transport mechanism of fabric OECTs must be revealed to fully understand the operation process.

### 3.2. Light-Emitting Diodes

A light emitting diode (LED) is a semiconductor device that emits visible light when electron hole recombination takes places inside the device. Since the first commercial infrared inorganic LED was presented by Holonyak *et al.* in 1962 [126], various LEDs have been developed around the world such as visible, ultraviolet, and infrared LEDs [127,128,129,130,131]. Since a solid semiconductor material is generally used to generate light in LEDs, LEDs were originally considered solid-state devices. A breakthrough occurred in 1987 when Tang and Van Slyke reported a new type of LED based on organic semiconductors [132]. Organic LEDs (OLEDs) have several advantages, including lower manufacturing cost, faster response time, potential flexibility, low power consumption, and less heat dissipation [133]. Since OLEDs are malleable, they can be compatible with flexible substrates and can be applied to wearable electronics. Current research into OLEDs focuses primarily on two-dimensional (2D) devices, however, one-dimensional (1D) OLEDs have begun to receive attention for future wearable electronics. In this section, various 1D fiber-based OLEDs will be discussed.

**Figure 6:**
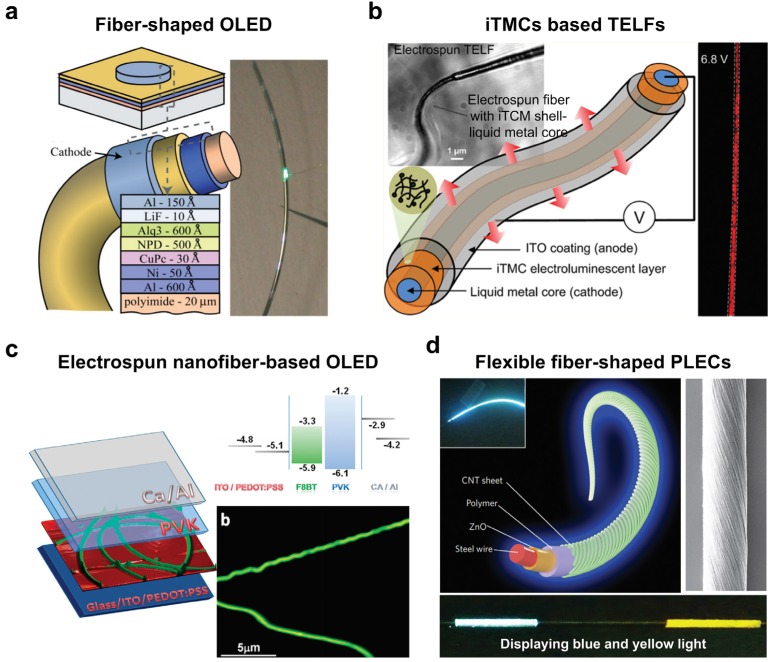
(**a**) Schematic illustration of a fiber-shaped organic light emitting diodes (OLED) structure compared with a typical OLED structure and a photograph of a flexed fiber with a green light-emitting pixel; (**b**) Schematic illustration and optical image of ionic transition-metal complex (iTMCs)-based electro-luminescent nanofibers (TELFs) fabricated via coelectrospinning and luminescence response of TELF under an applied voltage of 6.8 V; (**c**) Schematic of the conjugated polymer electrospun nanofiber-based OLED structure (left), energy level diagram (right top), and fluorescence microscopy image of an electrospun nanofiber, F8BT-PEO (right bottom); (**d**) Schematic illustration showing the structure of flexible fiber-shaped polymer light-emitting electrochemical cells (PLECs). Left inset: photograph of a fiber-shaped PLEC biased at 10 V, right inset: aligned CNT sheet wrapped around the modified stainless steel wire, and bottom inset: PLEC displaying blue and yellow light. Reproduced from [42,43,134,135] with permissions by John Wiley & Sons, Copyright 2007 and by ACS Publications, Copyright 2012, 2011 and by Nature Publishing group, Copyright 2015.

Connor *et al.* fabricated a fiber-based OLED using molecular organic compound as an active layer [134]. The inherently-flexible nature of molecular organic compounds is compatible with fiber structure. Figure 6a shows a schematic illustration of the fiber-based OLED structure used in this study [134]. Here, a polyimide-coated silica fiber was prepared as a substrate, and emission layers and organic charge transport layers were deposited between the anode and cathode electrodes via a vacuum thermal evaporation technique. A photograph of a 1 mm segment of green-light OLED on fiber clearly shows the emitting characteristics of green light. For a performance comparison of electricity and luminescence between a fiber-based OLED and planar OLED, they fabricated a planar OLED using a planar silicon/polyimide substrate and investigated the electrical and luminescence characteristics. The electrical and luminescence characteristics of fiber-based OLEDs showed comparable results to planar OLEDs; however, a slightly larger leakage current was measured at low bias due to the surface roughness of the fiber structure. In addition, the fiber is rather thick at ~480 μm, which can hinder practical use. Yang *et al.* recently fabricated single core-shell fiber OLEDs based on an ionic transition-metal complex (iTMC) using a coaxial electrospinning process [135]. A galinstan liquid metal core and a ruthenium tris (bipyridine) with poly (ethyl-oxide) (PEO) mixture shell were co-electrospun to from the cathode and electroluminescent layer, respectively. The anode was formed via thermal evaporation of indium tin oxide. Figure 6b shows a schematic illustration and optical image of an emitting iTMC [135]. The device was initially turned on at 4.2 V and bright electroluminescent was observed at 6.8 V. This work shows the possibility of direct fabrication of 1D flexible OLEDs without flexible substrates; however, the efficiency or brightness of the devices is not clearly shown.

Vohra *et al.* fabricated luminescent F8BT/PEO nanofibers using electrospinning [43]. Figure 6c shows a schematic illustration and fluorescence microscopy image of the conjugated polymer electrospun nanofiber-based OLED [43]. To fabricate the device, a thin layer of PEDOT-PSS was spin coated onto the ITO/glass substrates, and the F8BT/PEO nanofibers were directly collected onto the resulting layer. Then, a PVK (poly *N*-vinylcarbazole) solution was spin coated on top, and finally a thin layer of calcium and aluminum were evaporated. Here, the PVK-coated F8BT/PEO nanofibers functioned as an active layer. This work solved the charge injection issue in organic nanofibers by annealing the device at 150 °C, which changed the active layer into ribbon-like morphology. The measured external quantum efficiencies and luminance were 0.5% and 2300 cd·m^−2^ at 6 V, respectively.

Most recently, Zhang *et al.* developed a color-tunable and wearable fiber-shaped polymer light emitting electrochemical cell (PLEC), which allows for the integration of wearable LEDs in textiles [42]. The fiber-shaped PLEC was fabricated using an all-solution processing method, which increases the chance for practical use. Figure 6d presents a schematic diagram of a flexible fiber-based PLEC [42]. To fabricate the fiber-shaped PLEC, a stainless steel wire was dip coated with ZnO nanoparticles to form an electron transfer layer and decrease leakage current. Then, an electroluminescent polymer layer (a blend of PF-B, ethoxylated trimethylopropane triacrylate and lithium trifluoromethane sulphonate) was grafted over the ZnO-coated stainless steel wire, followed by wrapping the modified steel wire with the aligned carbon nanotube (CNT) sheet. The aligned CNT provided high electrical conductivity and flexibility in the device. Since the fiber-shaped PLEC is a 1D structure, the brightness was the same in all directions. In addition, the authors successfully integrated PLECs into fabrics and showed the tunable colors. This work shows promising benefits such as flexibility, light weight, high brightness that is maintained after bending, and tunable colors, as well as the possibility of integration into wearable electronic devices.

### 3.3. High Sensitivity Sensors

Sensors are widely used electronic devices which detect specific the characteristics of their surroundings and are present in a wide variety of applications such as electronics, aerospace, medicine, robotics, and machinery [136,137,138,139,140,141,142]. Types of sensors include temperature, pressure, humidity, gravity, sound, and vibration sensors [143,144,145,146,147,148,149,150]. In this review, we focus on textile-based pressure sensors, as sensing pressure is one of the most important functions of wearable electronics.

In 1996, a full-body, soft, flexible sensing suit was developed for a small robot by Japanese researchers [151]. They used an electrically-conductive fabric sheet and conductive strings with a spacer, and measured the contact resistance after applying pressure, demonstrating a promising remote robot sensor capable of controlling robotic body interactions. Shimojo *et al.* fabricated a tactile sensor using pressure-conductive rubber [152] via the development of a thin, flexible, attachable, tactile sensor that can be operated on curved surfaces. They measured the change in resistance under constant strain, observing a change in resistance during a period of 100 s. Their tactile sensor, when properly operated on a robot hand, showed good durability under repeated bending tests. Takamatsu *et al.* developed a large-area pressure sensor on a meter-scale using dye-coated conductive polymer (PEDOT:PSS) and perfluoropolymer dielectric film [153]. The sensor was constructed by weaving fibers into an area of 16 × 16 cm^2^. The sensitivity of this design ranged from 0.98 to 9.8 N/cm^2^, sensitive enough to detect human touch and therefore suitable for wearable keyboards and healthcare systems.

**Figure 7:**
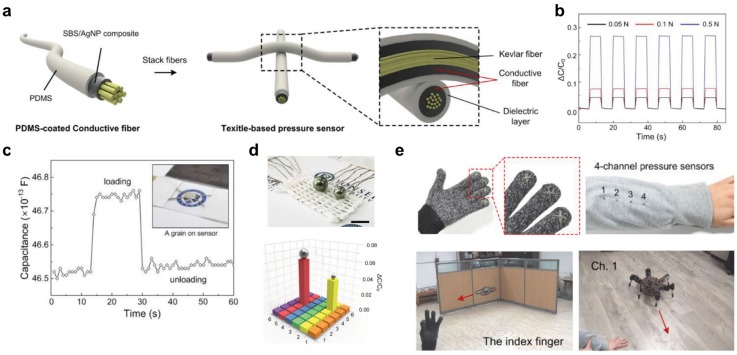
(**a**) Schematic structure of a textile-based pressure sensor fabricated with PDMS-coated conductive fibers; (**b**) Capacitive response of the pressure sensor for the various applied loads; (**c**) Capacitive response of the pressure sensor to the placing and removal of a grain (8 mg); (**d**) Fabrication of pixelated pressure sensor array; (**e**) Photographs of the smart glove and clothes with textile-based pressure sensors and operation using smart glove and clothes to control the drone and spider robot. Reprinted from [20] with permissions by John Wiley & Sons, Copyright 2015.

Recently, Lee *et al.* fabricated a textile-based pressure sensor using Ag-SBS composite conductive fibers [20]. A capacitive type of textile-based sensor was fabricated using a poly(dimethyl-siloxane) (PDMS) coating on the conductive fiber and cross-stacking two PDMS-coated fibers, as shown in Figure 7a [20]. Figure 7b shows the relative changes in capacitance of fiber-based pressure sensors via increasing loads of applied pressure [20]. In this operation, the conductive fiber-based pressure sensor showed stable responses to the various loads (Figure 7c), and detected the loading and unloading of grains of extremely low weight. This work further demonstrates e-textile applications with conductive fiber-based pressure sensors. The textile-based pressure sensor was pixelated through weaving techniques and detected the spatial distribution of external pressures, as shown in Figure 7d [20]. This textile-based pressure sensor was also sewn into a smart glove and clothes and showed the control of drone and spider robots, as shown in Figure 7e [20]. This work demonstrates that human-machine interfaces can be successfully operated using wearable textile-based pressure sensors with ultrahigh sensitivity and stability.

**Figure 8:**
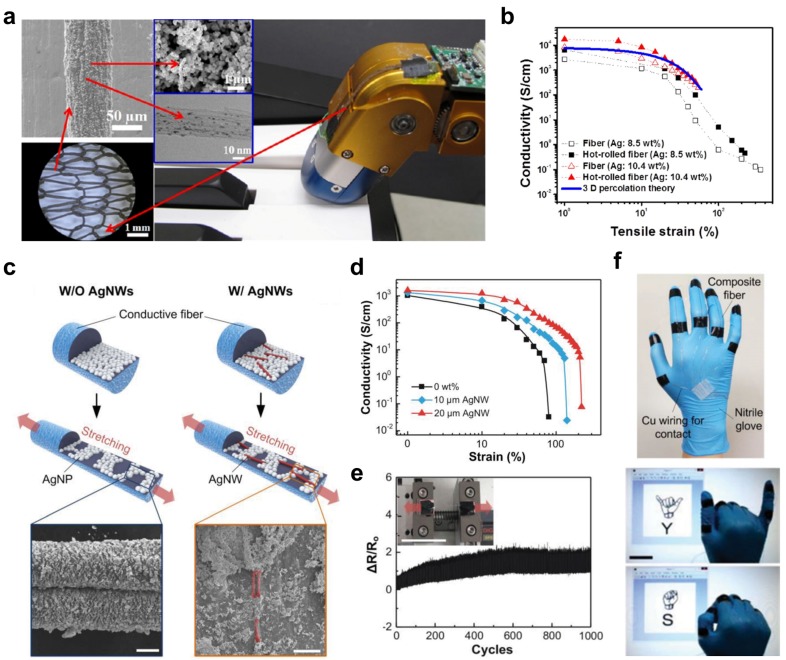
(**a**) FE-SEM and HRTEM images of knitted fabrics made from highly-conductive stretchable fibers. Bottom of this image shows an optical image of 2 ply rope. The knitted fabric was attached to a robot finger (as shown by arrow) for touch sensing; (**b**) Plot shows the conductivities of fibers as a function of tensile strain; (**c**) Schematic illustration of silver nanowires (AgNWs) and silver nanoparticles (AgNPs) in the composite fiber. (Left bottom image in navy colour line: SEM image of AgNP-mixed styrene-block-butadienstyrene (SBS) fiber without AgNWs at 50% strain and  and Right bottom image in red colour line: SEM images of the AgNW-AgNP embedded SBS fiber at 50% strain); (**d**) Changes in conductivity of the composite fiber depending on the length of the AgNWs; (**e**) Reliability test measured the changes in the normalized resistance of the composite fiber; (**f**) Photograph of a smart glove made with composite fibers and motion detection of English letters using a sign language. Reproduced from [90,154] with permissions by ACS Publications, Copyright 2014 and by John Wiley & Sons, Copyright 2015.

Ma *et al.* presented a new means of fabricating stretchable fabric [154]. To make stretchable knitted fabric, conductive stretchable fibers were first fabricated via wet-spinning. Figure 8a shows the surface morphology of conductive stretchable fibers composed of Ag nanoparticles, nAg-MWCNTs, and poly (vinylidene fluoride-co-hexafluoropropylene) (PVDF-HFP) matrix [154]. Stretchable fabric was then made by knitting the conductive stretchable fibers. The knitted fabric showed reversible characteristics under 100% tensile strain with mechanical and electrical stability (Figure 8b) [154]. In addition, the stretchable fabric was applied to a robotic finger system and demonstrated feasibility for practical application.

More recently, Lee *et al.* fabricated highly-stretchable conductive fibers composed of Ag nanowires (Ag NWs), Ag nanoparticles (Ag NPs), and SBS polymer using the wet spinning method [90]. Ag NWs allow for significant stretching capability in the fabric because they are aligned along the applied uniaxial strain and bridge the disconnected networks of Ag NPs inside the fiber, as shown in Figure 8c [90].

Figure 8d shows changes in conductivities with regard to various applied tensile strains [90]. As tensile strain increased, conductivities initially decreased, but improved again with increased strain. The reliability test showed stable operation under a repeatable bending test (Figure 8e) [90]. The authors further demonstrated the capability of detecting human motion using the fabricated stretchable sensor. The smart glove was integrated with fibers on each finger and successfully detected finger sign language. Figure 8f shows the detection of the letters “Y” and “S” via the bending of fingers [90].

## 4. Textile-Based Energy Applications: Energy Harvesting & Storage

Although commercial energy harvesting and storage devices using semiconducting thin film exhibit high performance, they also have limitations including their complex fabrication process and high cost. Recent novel materials investigated for energy related devices include dye, organic and inorganic polymers with high efficiency, large capacity, abundant material sources, easy fabrication, and flexibility. Along with the development of materials for energy devices, there are several studies on novel methods for energy harvesting using the human body, such as piezoelectric, triboelectric, and thermoelectric nanogenerators. Nanogenerators can provide self-powered systems that are useful in wireless devices. In particular, fiber-based nanogenerators have been primarily studied in the form of twisted, conjugated, or weaved multiple fiber electrodes integrated with supercapacitors.

### 4.1. Energy Harvesting from Human Motion

As the number of applications related to mobile electronic devices increases drastically, the development of long-lasting power sources for these devices is increasingly in demand. Sustainable self-powered sources have received significant attention as alternatives to conventional rechargeable batteries. Recently, energy harvesting from human body motions has proven a new means of operating many mobile electronic devices without the need for external power sources [155].

The piezoelectric effect is a unique ability of materials with polarization domains or non-centrosymmetric structures. These materials can generate an electrical charge in response to mechanical stresses such as compression, twisting, or distortion. One novel approach using the piezoelectric effect converts human mechanical motion into electrical energy. Wang *et al.* demonstrated that ultrasonic waves were converted into electricity using a Pt-coated zigzag electrode with vertically-aligned ZnO nanowires (NWs) [156]. They optimized the size and shape of the ZnO NWs and obtained high-output power per unit of area reaching ~10 µW/cm^2^. The generated energy can potentially facilitate long-term sensors that are applicable to mobile electronic devices and systems [157]. Zhang *et al.* reported a flexible nanogenerator (FNG) fabricated using BaTiO_3_ nanowire and polyvinyl chloride (PVC) composite piezoelectric fiber [158]. After optimizing their process parameters, they placed FNG on a bent human arm, producing voltage and current outputs up to 1.9 V and 24 nA, respectively. Several methods have been developed for harvesting electrical energy by converting mechanical energy into electricity using piezoelectric materials [159]. A prototype electrostatic non-resonant generator has been designed and optimized by Mitcheson *et al.* [160]. Using flexible polymer membranes, they can generate an output voltage of 250 V when the generator is pre-charged to 30 V. Qin *et al.* have described the concept of textile-based piezoelectric nanogenerators that can harvest electrical energy from the friction between two fibers [161]. Although only a small amount of output current (~±5 pA) was obtained during the pulling/releasing cycles of the nanogenerator, they established a novel method of energy harvesting using fabrics.

**Figure 9:**
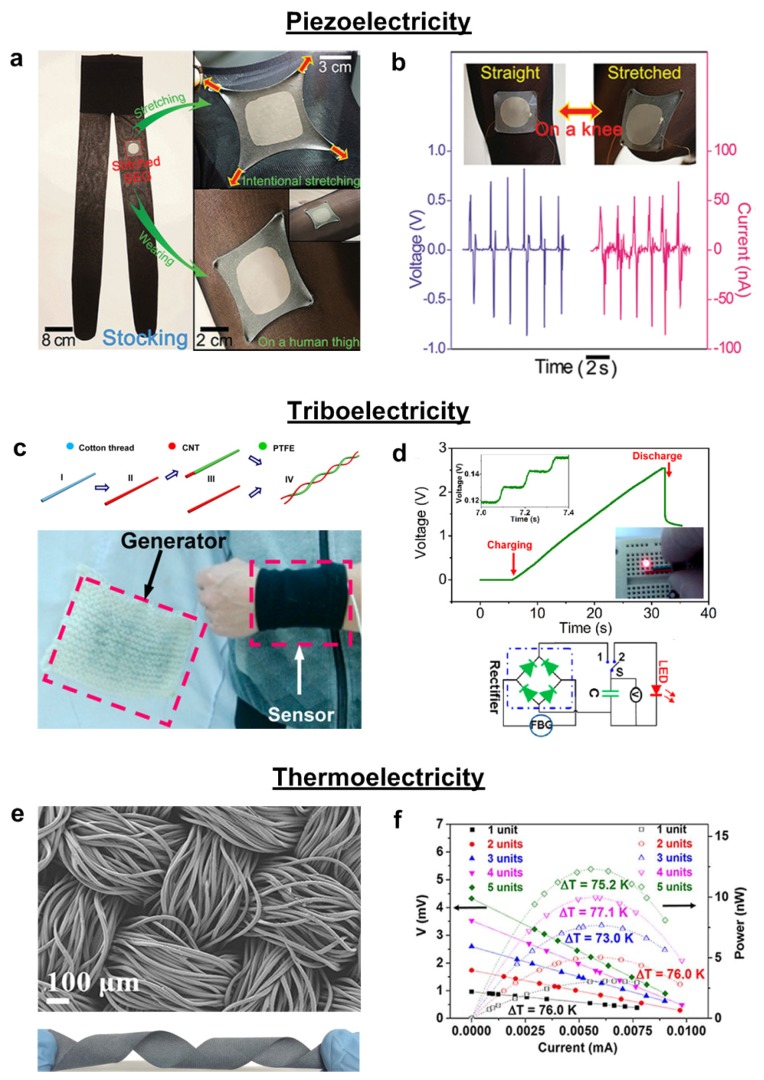
(**a**) Digital photographs of the stretchable elastic-composite generator (SEG) stitched on a nylon stocking. Photograph of the SEG intentionally stretched and fitted over a human thigh; (**b**) Stretching on human knees and the generated voltage and current from the SEG on the stocking by bending and straightening the knee; (**c**) Schematic diagram illustrating the detailed fabrication process of a fiber-based generator (**above**) and woven fabric FBG sewed on a lab coat (**below**); (**d**) The voltage charging curve of the 2.2 μF commercial capacitor by the “power shirt” and typical bridge rectifier circuit for transforming alternating current to direct current; (**e**) SEM image (**above**) and digital photography (**below**) of the polyester fabric after PEDOT:PSS coating; (**f**) Experimental results of output voltage and power as a function of current for the fabricated devices. Current–power curves are parabolic for all devices. Reproduced from [162,163,168] with permissions by John Wiley & Sons, Copyright 2015 and by ACS Publications, Copyright 2014 and by Nature Publishing Group, Copyright 2014.

Further, Jeong *et al.* reported a novel approach to fabricating a high-performance hyper-stretchable nanogenerator (SEG) with extremely long Ag NWs [162]. The hyper-stretchable piezoelectric elastic composite (PEC) rubber polymer was composed of very long nanowire percolation (VLNP) electrodes and a well-dispersed mixture of lead magnesioniobate lead titanate (PMN-PT) particles and multi-walled carbon nanotubes (MWCNTs). The composite exhibited high stretchability over 200% strain without any mechanical cracking; this could be attributed to variation in the piezoelectric dipoles in the PEC caused by stretching, and helps accumulate electrons, generating voltage and current. An open-circuit voltage (~4 V) and short-circuit current (~500 nA) were obtained and the generated energy can sufficiently operate commercial electronic units. The SEG was tested in biological motion after being sewn onto the knee of a stretchable stocking, as shown in Figure 9a,b [162]. However, textile-type energy harvesters seem to face some prevalent problems such as low energy conversion efficiency, wide spectra of vibration frequencies, and time-dependent amplitudes.

Recently, triboelectric nanogenerators (TENG) have been developed that can harvest electrical energy from mechanical friction, exhibiting high performance, a simple fabrication process, cost-effectiveness and green technology [163,164,165,166]. The principle of TENG operation is the combination of triboelectric and electrostatic induction [166,167]. When two different polymer surfaces are rubbed or compressed, opposite sign electrostatic charges are generated on each surface of the polymer films. Due to the formation of a dipole layer, triboelectric potential in TENG is generated and drives electrical charges through the external load, resulting in the generation of an open circuit voltage and a short circuit current. Lee *et al.* reported a textile, substrate-based triboelectric nanogenerator (T-TENG) fabricated using an assembly of Au-coated fabric and polydimethylsiloxane (PDMS) [165]. To enhance the surface friction energy, thermally-evaporated Al nanoparticles (NPs) were conjugated with Au-coated textile top electrodes. Triboelectric energy was generated when two textile electrodes were repeatedly compressed and released. The validation of T-TENG in energy harvesting using human motion was further examined on a human arm. To utilize this device in practice, they demonstrated that the alternating current (ac) output signals obtained from the T-TENG were converted to direct current (dc) using a bridge rectifier. Using the rectified dc current, a commercial capacitor was charged and a light-emitting diode (LED) was turned on. When the T-TENG attached to the human arm was bent and released, the generated output voltage and current were 139 V and 39 μA, respectively. The high efficiency can be attributed to enhanced surface roughness induced by uniformly-distributed Al NPs on the textile electrodes.

The abovementioned nanogenerators were produced on flexible, metallic-based electrodes. However, to realize the true potential of wearable electronics, it is necessary to study textile-based flexible energy harvesters without metal electrodes. Metal-free, fiber-based TENG capable of converting biomechanical motion into electricity were introduced by Zhong *et al.* [163]. The TENG were produced using two kinds of pretreated cotton threads, each coated with multi-walled carbon nanotubes (CNT) and polytetrafluoroethylene-carbon nanotubes. These two fibers were entangled with each other to form a light-weight and flexible fiber-based generator (FBG). When the entangled fibers were stretched, the inter-fiber gap was reduced and the CNT layer on the CNT-coated cotton thread (CCT) became positively charged due to induction, creating free electrons. Woven FBG was applied to a “power shirt” with a wireless temperature sensor capable of charging to a capacity of 10 nF, as shown in Figure 9c [163]. Figure 9d shows that the rectified DC voltage obtained from the FBG was 2.4 V with the charging time of 27 s, showing that the FBG is capable of lighting a red LED. Since the fabricated power shirt can monitor body temperature cost-effectively due to the self-powered TENG system, it makes viable the possibility of woven textiles in the field of mobile medical systems.

Recently, interest in thermoelectric materials has increased due to their ability to generate energy from waste heat. In the presence of temperature gradients, charges in the thermoelectric material are diffused from the hotter to the colder side, inducing electrochemical potential inside the material. In wearable electronics, fiber-based thermoelectric generators (TEGs) are a unique energy harvesting device converting body heat into a reliable power source [168,169,170]. Since human body heat is a steady, plentiful, and convenient source of heat, it is meaningful and useful that TEG can harvest waste body heat. Kim *et al.* described a wearable TEG utilizing a combination of fabric and printed thermocouples that can be easily embedded in clothing to harvest low-temperature energy [169]. To prevent skin irritation, polymer-based fabric was used as a substrate and a conductive silver thread was used for electrical connection. When the TEG was integrated in a shirt, the temperature of the skin was maintained at 32 °C under cold (5 °C) or hot (25 °C) environments. Interestingly, the output power under cold environment was significantly higher at ~147 nW than that of the hot ambient environment, at ~8.1 nW.

To investigate the suitability of thermoelectricity in wearable power generators, Du *et al.* reported a textile-based TEG featuring a long operating time, easy maintenance, and high reliability [168]. This TEG overcame the common problems of toxicity, poor processing ability, and impermeability by introducing a PEDOT:PSS conducting polymer. The inset of Figure 9e shows the morphology of polyester fabric coated with PEDOT:PSS, which had excellent flexibility in any shape [168]. The PEDOT:PSS-coated fabrics were connected using silver wires, as shown in Figure 9f. Using multiple strips of the fabricated device, the output voltage was improved to 4.3 mV due to the Seebeck effect. Using a fabric thermoelectric generator containing 5 thermoelectric strip units, the maximum output of electrical power (*P*_max_) was 12.29 nW at a Δ*T* = 75.2 K, as shown in Figure 9f [168].

Despite the various advantages of TEGs, their limitations include a low energy conversion efficiency rate, slow technology progression, limited application possibilities, and the requirement of a constant heat source. The fabrication process is also complex because TEGs should be flexible and tightly adhere to the human body.

### 4.2. Solar Cells on Textiles

In the past decades, next-generation solar cells such as dye-sensitized solar cells (DSSC), organic solar cells (OSC), and perovskite solar cells (PSC) have attracted much attention due to their low cost and high efficiency [171,172,173]. These solar cells are based on light absorption using dye, organic, or perovskite materials that generate mobile electron-hole pairs [174]. The generated electrons and holes are separated and collected into cathodes and anodes, respectively. Recently, fiber-based solar cells have shown great potential for use in textile electronics and represent a new direction in solar energy harvesting since they can be woven into various types of flexible structures at low-cost. However, these devices would benefit from further study into the development of facile fabrication methods for large-scale production and realization of actual products as well as the feasibility of integration in cloth. Several researchers reporting wearable solar cells have primarily focused on inorganic, dye-sensitized, perovskite, and polymer-based solar cells with lightweight, flexible, and ease of manufacture [38,99,175,176].

**Figure 10:**
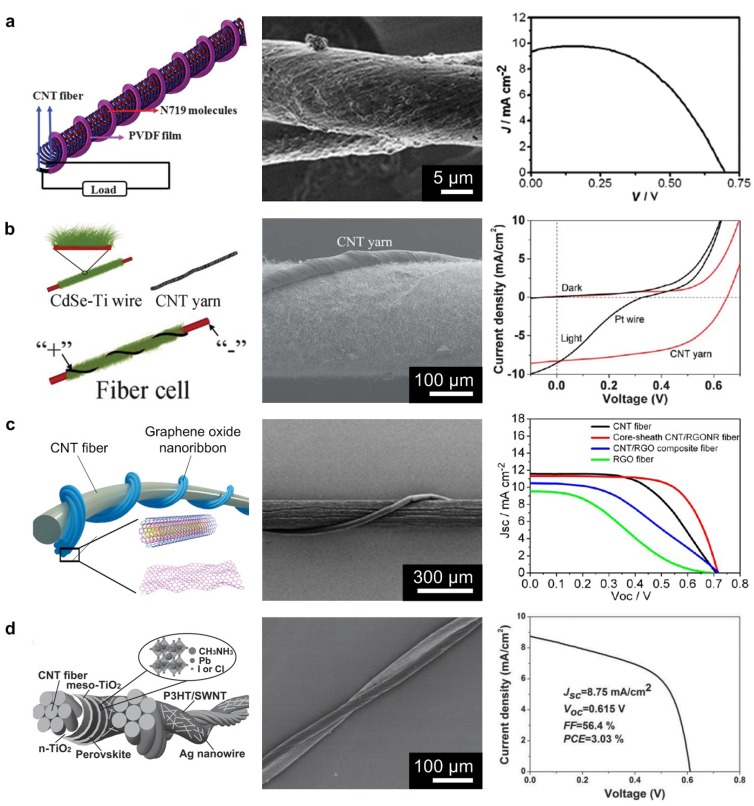
(**a**) Schematic (**left**) and SEM image (**middle**) of the twisted CNT/N719 working electrode and carbon nanotube/poly-vinylidene fluoride (CNT/PVDF) counter electrode. A typical density–voltage (J–V) curve (**right**) with improved performance after modification of the working electrode; (**b**) Schematic fabrication process (**left**) and SEM image (**middle**) of photo electrochemical cells with a primary electrode of Ti wire, light absorption layer of CdSe nanowire, and counter electrode of CNT. J–V curves under dark and light conditions of different fiber cells using a CNT yarn and Pt wire counter electrode; (**c**) Schematic (**left**) and SEM image (**middle**) of wire shaped dye-sensitized solar cells with twisted CNT fiber. Current density-voltage curves for a wire-shaped DSC (**right**); (**d**) Schematic diagram of double twisted solar cell supported by the CNT fiber and the chemical structure of the perovskite material (**left**). The SEM image shows twisted fibrous solar cells (**middle**). The current J–V curve is shown for electrical performance (**right**). Reprinted from [38,99,175,178] with permissions by Royal Society of Chemistry, Copyright 2012, 2012 and by ACS Publications, Copyright 2013 and by John Wiley & Sons, Copyright 2015.

Recent studies on dye-sensitized photovoltaic fibers have used primarily copper, steel wire, or polymer fibers coated with an indium tin oxide (ITO) electrode [177,178]. Cai *et al.* demonstrated a new method of fabricating 0.3 mM cis-bis(isothiocyanato)bis(2,2ʹ-bipyridyl-4,4ʹ-dicarboxylato)-ruthenium(II) bis-tetrabutylammonium (N719)-absorbed CNT fibers as a working electrode and CNT fibers coated with poly-vinylidene fluoride (PVDF) as a counter electrode for dye-sensitized photovoltaic application [178]. The fiber-based solar cell was integrated into textiles via conventional weaving techniques. The left side of Figure 10a shows a schematic illustration of the photovoltaic wire, while the middle image in Figure 10a shows an SEM image of the twisted CNT/N719 and CNT/PVDF fibers [178]. The open-circuit voltage and short-circuit current density of the fiber-based dye-sensitized solar cell were 0.69 V and 9.84 mA·cm^−2^, respectively, with a power conversion efficiency of 3.90%, as shown in the right image in Figure 10a.

Zhang *et al.* demonstrated CdSe NWs and CNT yarn-based photovoltaics, with power conversion efficiency up to 3% [179]. The left image in Figure 10b presents a schematic illustration of fiber-based CdSe-grafted Ti wire fabricated via chemical vapor deposition (CVD) with twisted CNT yarn. The SEM image in Figure 10b shows that the twisted CdSe-Ti wire and CNT fiber make smooth contact without the addition of electrolyte due to the flexibility of the yarn. The performance of CNT yarn and CdSe-Ti wire heterojunction with polysulfide electrodes was characterized by measuring the J-V curve of the fiber cells. The open-circuit voltage (*V*_oc_), short-circuit current density (*J*_sc_), fill factor (*FF*), and efficiency were 0.65 V, 8.25 mA·cm^−2^, 55%, and 2.9%, respectively.

Recent successes in perovskite (CH_3_NH_3_PbX_3_, X = Cl, Br, I) solar cells provide encouragement for flexible solar cells in wearable electronics [180,181]. Perovskite materials have many attractive properties including broad spectral absorption and high carrier mobility. The efficiency of thin film photovoltaic cells using perovskite materials have been recorded at up to 20% [182,183]. Some attempts have been made to fabricate fiber-based perovskite solar cells for wearable electronics [38,184]; however, it is difficult to integrate the fibers into clothes due to the insufficient flexibility and low surface area of perovskite materials. To solve this issue, Li *et al.* described a CNT fiber-supported double-twisted perovskite solar cell featuring high stability where the perovskite nanocrystals on the CNT fiber sustain the interfacial adhesion under stress [38]. The left image in Figure 10d shows a schematic illustration of the CNT fiber-supported double-twisted perovskite solar cell using twisted *n*-TiO_2_ added CNT, meso-TiO_2_, CH_3_NH_3_PbI_3−*x*_Cl*_x_*, P3HT/SWNT, and an Ag nanowire network [38]. The P3HT/SWNT was used as a hole transport layer, and the Ag nanowire was added to increase the surface area between electrodes. The fabricated perovskite solar cell fiber can be seen in the middle of Figure 10d; the optimized maximum power conversion efficiency (PCE) was 3.03%. Although the performance of the abovementioned textile-based photovoltaic harvesters are, some limitations such as mass production, high cost, and low energy conversion efficiency remained as obstacles to utilization in real fabrics. Further research into textile-based solar cells is needed for the realization of future wearable solar cells.

### 4.3. Fabric-Based Supercapacitors

Supercapacitors (SCs) have attracted significant attention as outstanding storage components due to their high power density, operational safety, long cycle life, and fast charge-discharge rates [185,186,187]. Depending on the mechanism of charge storage, SCs can be categorized into two types: the Electrical Double Layer Capacitor (EDLC) and pseudocapacitor. EDLCs are typically fabricated using two electrodes separated by a carbon-based electrolyte and an electrical double layer is formed between the electrodes and electrolyte based dielectric. Pseudocapacitors function based on reversible faradaic-type charging and discharging. The supercapacitor is made of conducting polymers and transition metal oxides, and the mechanism of energy storage depends on the redox reactions between materials. To integrate these supercapacitors into textiles, their components, including electrodes and current collectors, will have to be flexible.

**Figure 11:**
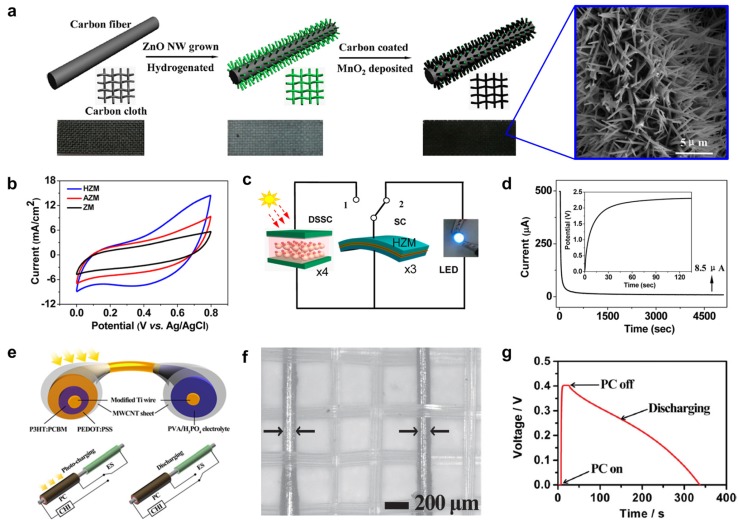
(**a**) Schematic fabrication process and SEM image of a hydrogenated single crystal ZnO (HZM) and amorphous ZnO-doped MnO_2_ core-shell electrodes; (**b**) Current-potential characterization with different electrodes with scan rates of 100 mV/s; (**c**) Design of a stand-alone self-powered system consisting of dye-sensitized solar cells (DSSCs), SCs, and LEDs; (**d**) Leakage current characteristics of three SCs in series and voltage profile (inset) charged by four DSSCs in series; (**e**) Schematic illustration and circuit connection mechanism in the process of charging and discharging of photovoltaic conversion (PC) and energy storage (ES) with titania nanotube-modified Ti wire and aligned MWCNT sheet as two electrodes; (**f**) Photography of energy fibers woven with each other to form flexible textiles; (**g**) Charging-discharging curve with a current of 0.1 μA during the discharging process with the ES part. Reproduced from [188,191] with permissions by ACS Publications, Copyright 2013 and by John Wiley & Sons, Copyright 2014.

Recently, Yang *et al.* demonstrated a pseudocapacitor-based supercapacitor with improved redox reaction and good reversibility [188]. To further improve the performance, one study introduced a hybrid architecture using MnO_2_-coated hydrogenated zinc oxide nanowires (HZnO) grown on a flexible carbon cloth [189,190]. High electrochemical performance was exhibited by amorphous ZnO-doped MnO_2_ core-shell nanocable (HZM) electrodes with a polyvinyl alcohol (PVA)/LiCl neutral electrolyte. Figure 11a shows a schematic illustration of the synthesis process of core-shell structures and an SEM image of HZM. The cyclic voltammetry (CV) measurement shown in Figure 11b indicates that the HZnO electrode had a higher free carrier concentration and better electrical stability when compared to untreated ZnO and air-annealed ZnO electrodes which is attributed to the large surface area of the HZnO electrode.

This improvement was further explained by the insertion of hydrogen acting as a shallow donor for the neutral and electrically-inactive oxygen vacancies in *n*-type ZnO. Figure 11c shows the combination of the energy harvesting module, dye-sensitized solar cells (DSSCs), and energy storage component that could illuminate a commercial blue LED from charged SCs when the solar source is off. Figure 11d shows a significant initial drop in the leakage current; this measurement stabilized after 4500 s, indicating the presence of few impurities in the electrode and electrolyte. This research contributed to both the fundamental advancement of this technology as well as the advancement of potential of SCs applications.

Zhang *et al.* also developed a flexible energy fiber composed of titania nanotube-modified Ti wire and MWCNT that can store energy as well as harvest solar energy [191]. P3HT: 1-(3-methoxycarbonyl) propyl-1-phenyl [6,6]C_61_ (PCBM) and PEDOT:PSS with MWCNTs were used as photoactive materials and a PVA/H_3_PO_4_ gel electrolyte was utilized for capacitor storage, as shown in Figure 11e. When the P3HT: PCBM heterojunction generates excitons and separate carriers (electrons and holes), the carriers are transported through MWCNTs and PEDOT:PSS, and then stored in the energy storage area. Figure 11f shows the energy fibers that were successfully woven into textiles. Figure 11g describes the charge-discharge curves, which indicate a fast charging process. The maximum efficiency and energy storage capacity of the fibers are ~1% and ~65%, respectively. These advances represent various new opportunities for the future of wearable electronics.

## 5. Current Limitations of Wearable E-textiles

Aforementioned discussion on the wearable E-textiles presents them as a good futuristic candidate in daily life of human outfits. However, for the true realization of wearable E-textiles, some basic issues must be addressed.

One of the main limitations of wearable E-textiles which require more concern is toxicity due to hazards materials used in electronic components. If, we integrate the electronic components in the wearable textiles they must be safe and non-toxic. Electronic components contain toxic material for example batteries have toxic and liquid electrolyte material like Li-ion batteries and tetraethylammonium tetrafluoroborate in acetonitrile [186,192]. Therefore, garments integrated with these electronic components are not suitable to wear because they will be in directly contact with skin. To resolve this issue some reports are available to use a non-toxic material. Jost *et al.* have fabricated flexible and durable textile electrodes for supercapacitors by using non-toxic electrolyte material (sodium and lithium sulfate) [193]. The as prepared fabric electrodes have shown high areal capacitance but the problem with these fabric electrodes is the requirement of an integrated device to seal the liquid electrolyte to avoid its leakage. Further, a non-toxic electrolyte based wearable supercapacitor has been suggested by pasta *et al.* in their well-arranged study [194]. They have reported a simple fabrication technique for supercapacitor showing high specific capacitance and cycling stability. But the drawback of as prepared supercapacitor is the hydrogen evolution overvoltage prevents the operative voltage range as similarly reported by Prosini *et al.* [195]. Several other reports are also available on non-toxic electrolyte materials. However, further improvements are required in order to use these components in wearable textiles and toxicity issue should be carefully monitored before employ them in wearable fabrics.

Other drawback of these wearable E-textiles is related to their multiple uses, *i.e.*, washability and durability. To the best of our knowledge, there is no study on multiple washable textiles integrated with electronic components. In general, multiple washing and special care is required to worn these garments in daily life. Also, the long term usage of these wearable E-textiles is again a matter of concern. It is required to know that the repairing as well as exchanging of electronic device is possible during their long term use by wearer. The other challenge for researchers is to focus on the packaging of electronic components and device performance for their usage over the longer time. Due to crack and poor bending properties of fiber–based electronics, failure of packaging is observed [196]. These wearable electronic fabrics must hold their sensing, actuating and responding properties in qualitative way during their everyday wear and off-course maintain comfort fit to human body. This can be achieved by replacing hard printed circuit board to flexible electronics.

For the practically implementation of these wearable fabrics the mass production is required and for this an interdisciplinary work must be involved between electronic as well as textile peoples. But to the best of knowledge there is a lack of combination of electronics and textiles industries. Thus, for future developments of E-textiles it is required that the experts of these two firms must work together. Other issues include the biocompatibility of these textiles, development of sophisticated multi-functionalities, stable performance of devices and light weight fabrics for future applications. Thus, any realistic commercial implementation can be achieved after addressing all pointed issues.

## 6. Conclusions and Perspectives

In this review article, a brief overview of textile electronics is presented based on electronic components, in which the various types of conductive fibers and fabrication techniques are summarized using different materials such as polymers, CNTs, graphene, and metal nanowires. Fiber-based electronic components (transistors, LEDs, and sensors) have also been described. Furthermore, textile-based energy harvesting and storage devices were explained for energy applications in textile electronics. Although considerable performance in textile-based electronic devices has already been achieved, further efforts to improve performance are necessary. A significant body of theoretical and experimental research has been carried out to understand the mechanism and characteristics of E-textiles in wearable electronics. To commercialize textile-based devices in wearable electronics, some important issues will have to be addressed, including mass production, integration into clothes, non-toxic technology, and long-term usage. If the E-textiles can overcome the aforementioned issues, a new era in wearable electronics will begin.
